# Evidence of areca nut consumption in the United States mainland: a cross-sectional study

**DOI:** 10.1186/s12889-022-13262-1

**Published:** 2022-05-07

**Authors:** Irene Tami-Maury, Suzanne Nethan, Jessy Feng, Hongyu Miao, George Delclos, Ravi Mehrotra

**Affiliations:** 1 The University of Texas Health Science Center at Houston School of Public Health, Houston, TX USA; 2 The University of Texas MD Anderson Cancer Center, Houston, TX USA; 3 National Institute of Cancer Prevention & Research, Noida, India; 4 University of Texas Rio Grande Valley School of Medicine, Edinburg, TX USA; 5 India Cancer Research Consortium, Delhi, India

**Keywords:** Areca, Betel, Prevalence, US mainland, Asian immigrants

## Abstract

**Background:**

Areca nut (AN) is an addictive substance consumed in the Southeast region and is highly associated with oral premalignant lesions and oral cancer. The impact of AN use in the United States (US) is largely unknown, but the products are readily available and probably used by a significant fraction of Asian immigrants or descendants living in the US. We aimed at assessing AN use prevalence among the Asian community in Houston, Texas.

**Methods:**

A cross-sectional questionnaire was used to interview adult individuals (≥ 18 years of age) who self-identified as Asian immigrants or descendants residing in Houston. Means, frequencies, and proportions were reported. Factors associated with AN use were evaluated using logistic regression.

**Results:**

We surveyed 275 individuals (58% women, 43% between 35–54 years old, 67% born outside of the US, and 6% concurrent smokers). Among respondents, 91% were familiar with AN products, 17% self-reported ever use of AN products in the US, and 31% had friends/family members who were AN ever users. AN use was significantly associated with being Indian Subcontinent immigrants or descendants (ISID) (OR = 3·9; CI: 1·10,13·81; *p* = 0·035) and having friends/family members using AN products (OR = 6·2; CI: 1·69, 22·69; *p* = 0·006).

**Conclusions:**

Our findings provide quantitative data on the prevalence of AN ever use and context for future AN prevention and cessation interventions specific to the Southeast Asian groups living in the US mainland. This is crucial for the prevention and control of oral cancer and other detrimental conditions related to AN consumption.

## Background

Areca nut (AN), the *Areca catechu*palm seed, is the fourth most widely consumed addictive substance in the world after tobacco, alcohol, and caffeine [1]. While AN consumption is particularly prevalent in Southeast Asia, where it is widely grown [2, 3], no further attempts have been made to assess the prevalence estimates of AN use worldwide.

For many Southeast Asian countries, AN chewing is an ancient practice ingrained in the culture [4]. However, AN is a proven independent carcinogen [5], and the addition of tobacco further increases the risk of developing oral cancer. A high incidence of oral cancer has been noted among many Southeast Asian countries, including India, Sri Lanka, Myanmar, Thailand, southern China, and Taiwan, where chewing AN is a common practice [6].

AN can be consumed fresh, sliced, dried, or fermented, combined with slaked lime, catechu, spices (e.g., cardamom, saffron, cloves, aniseed, turmeric, mustard, etc.) with or without tobacco in small home-made pouches. AN can also be found in commercial packages (sachets) and tins widely available for purchase. The two most popular AN presentations in the Indian Subcontinent, *'Gutkha'* and *'Pan Masala,'* are also exported to other countries, including the United States (US). *Gutkha* is a combination of AN, slaked lime, catechu, spices, and powdered tobacco, whereas *Pan Masala* comprises all these ingredients but tobacco. *Pan Masala* should not be confused with *Paan*, which is a non-commercial preparation with multiple ingredients such as AN, slaked lime, various spices (cardamom, cloves, turmeric, saffron, aniseed, mustard), sweeteners, and tobacco (optional), wrapped in a betel leaf [5].

The increasing globalization and human migration have allowed AN products to become readily available outside of Southeast and South Asia [7]. The US Food and Drug Administration (FDA) has recognized the carcinogenic properties of AN [8]. Furthermore, the harmful effects of AN use with the development of oral submucous fibrosis, a type of oral potentially malignant disorders (OPMD), has been mentioned in the 2000 US Surgeon General's report on oral health in America [9]. Despite this, the import and sales of AN products are not strictly controlled or specially taxed, representing a serious health risk to consumers in the US [10, 11].

Within the past 25 years, only four reports about AN consumption in the US mainland were found in the scientific literature. A study published in 1994 and conducted [12] among ten interviewed Cambodian refugee women who were AN users revealed that AN products are readily available in Asian markets in San Diego, California. A subsequent study conducted in 2006 in New York City with a sample of 138 participants found 67% and 77% of current *Gutkha*use among Bangladeshi and Indian-Gujarati immigrant adults, respectively [13]. In the same study, Bangladeshi and Indian-Gujarati immigrant adults reported 70% and 5% of current *Pan*use, respectively. Another report published in 2008 showed that AN raw ingredients, commercial packets, boxes, or bags were available for purchase in 5 Asian grocery stores visited by the research team in Richmond, Virginia [14]. A more recent research effort conducted in Clarkston, Georgia, reported 65% AN use prevalence among a sample of 59 individuals who were familiar with AN products. Most of these individuals were refugees migrating from South and Southeast Asian countries [15].

Houston is considered the fourth most populous city in the US. This vast metropolis covers Harris County primarily, with small portions of the city extending into Fort Bend and Montgomery counties. The Asian population in Harris County has increased from 2·0% in 1980 to 7·7% in 2010, becoming the fastest-growing racial and ethnic group in Houston. The majority of the Asians in Houston is comprised of the Vietnamese (32·0%), Indian/Pakistani (20·0%), Chinese/Taiwanese (17%), Filipino (9·0%), and Korean (5·0%) populations [16]. As the Asian population continues to grow in Houston and other cities across the continental US, it is crucial to learn more about AN consumption and its availability. This information will be critical for developing and implementing effective educational and awareness interventions to prevent and control oral cancer and other conditions associated with AN use among these vulnerable groups. Therefore, the purpose of this cross-sectional study was to assess AN use prevalence and AN purchase patterns among South and Southeast Asian immigrants and descendants living in Houston, Texas.

## Methods

### Study population and design

The study population was comprised of adults (≥ 18 years old) Asian immigrants or descendants living in in Harris County, Texas [17]. Potential participants were recruited by convenience sampling, in an arbitrary manner, at local community events and health fairs targeting Asian populations between June 2018 and June 2019. Graduate research assistants approached potential respondents and invited them to participate in the study. Respondents were informed that completing the survey indicated their willingness to participate in the study. The survey research was conducted anonymously, and participation was voluntary. Respondents had the right to terminate their participation at any time. They were also informed that collected data would be reported only in aggregate form, in a manner that did not allow individual responses to be identified. There was no compensation for participating in this research effort. An expected sample size of 384 respondents was based on the following parameters: population size of 249,853 Asian individuals in Harris County [17], 5% absolute accuracy, and 95% confidence level. All research activities were performed in accordance with relevant guidelines and regulations (Declaration of Helsinki). Participation was voluntary, and written informed consent was waived according to approved survey protocol. The study protocol was approved by the Institutional Review Board of The University of Texas MD Anderson Cancer Center (Protocol #PA17-0804) and The University of Texas Health Science Center at Houston (Protocol #HSC-SPH-20–0221).

### Data collection

The cross-sectional data collection instrument was administered as an interview-based questionnaire (23 items). All interviewers completed training in Human Subjects Research and attended an additional one-hour meeting to discuss the questionnaire's items and methodology before initiating data collection.

The paper-and-pencil anonymous questionnaire was administered in English, as well as in several Asian languages such as Urdu, Hindi, traditional Chinese, simplified Chinese, and Vietnamese, which are predominantly spoken among the Asian immigrants/descendants living in Houston. A certified translation was essential to affirm that the questionnaire was translated to the above languages accurately. Interviewees with limited English proficiency were asked about their language of preference by presenting them with a one-page language identification card with the sentence “I speak [LANGUAGE]” in Urdu, Hindi, traditional Chinese, simplified Chinese, and Vietnamese. Once the language was selected, the potential study participant was redirected to an interviewer who was fluent in such language.

Since participation in the study was voluntary and the anonymous questionnaire involved minimal risk, written informed consent was waived by the Institutional Review Board of The University of Texas MD Anderson Cancer Center (Protocol #PA17-0804). The first section of the questionnaire recorded sociodemographic information such as gender, age range, level of education (recoded into two mutually exclusive groups: High school or less *vs.* Higher than high school), employment status, place of birth (for those foreign-born, two additional questions addressed country of origin and age at US immigration), and language(s) spoken at home. The second section was a self-perceived assessment of health status, which included a history of cancer diagnosis, if any. The third section corresponded to self-reported cigarette smoking. Participants who responded '*Yes'* to the questions: *'Have you smoked at least 100 cigarettes (5 packs) in your entire life?'* and *'Do you now smoke every day or some days?,'* were classified as current smokers. Non-current smokers were respondents who had smoked at least 100 cigarettes in their lifetime but had quit smoking at the time of questionnaire completion or had never smoked. The last section collected information about AN use and products. Study participants were asked if they were familiar with AN products (a list of names and pictures of AN products were included in the questionnaire). They were also asked about the availability of the AN products (e.g., at regular US grocery stores, US health food stores, Asian stores in the US, online, at their home, at friends'/relatives' homes, elsewhere in the US, etc.). This section also included questions about participants' perception of health risks associated with AN use, self-reported AN consumption while in the US, as well as AN use among family members and/or friends living in the US. AN ever users were also asked about their age of initiation.

Before collecting data in the field, interviewers learned about the research protocol, the importance of the study, and the rationale behind the inclusion of each question in the data collection instrument. They were also trained to introduce the study to potential participants and rehearsed data collection procedures. The interviewers were research assistants members of the study team with formal training in human subjects research and enrolled in graduate programs in health professions and/or public health.

The questionnaire took approximately 7 min to complete, and no incentives were provided to the participants. Data collected through the paper-and-pencil questionnaires were transferred to an electronic dataset using the Qualtrics platform. Subsequently, data were converted to SAS for analysis.

### Statistical analysis

Since a substantial proportion of the world's population engaged in AN use lives in the Indian Subcontinent, and that immigrant and descendants from this region represent the second-largest Asian group in Houston, two ancestry subgroups were computed for analysis purposes: Indian Subcontinent immigrants or descendants (ISID) and the rest of the study sample (Non-ISID). ISID were those individuals who either migrated to the US from Bangladesh, Bhutan, India, Maldives, Nepal, Pakistan, and Sri Lanka, or reported speaking at home any of the Indian Subcontinent languages such as Hindi, Gujarati, Urdu, among others.

Chi-square test (or Fisher exact test when appropriate) was used to compare all categorical variables between ISID and Non-ISID, while *t*-test was used to compare continuous variables.

Being ISID was used to predict AN ever use using logistic regression, adjusting for sociodemographic determinants such as age, gender, place of birth, education level, employment status, as well as smoking status and having family members and/or friends ever using AN products. The significance threshold was set at 0.05. All analyses were conducted using SAS v. 9·4 (SAS Institute Inc., Cary, NC, USA).

## Results

A total of 275 Asian descendants or immigrants residing in Houston agreed to participate in the study. More than three-quarters of the respondents (76·0%) were between 18 and 54 years old, with more than half of the sample being females (58·3%). The majority of the study participants had a greater than high school education level (79·6%) and were employed (71·1%) by the time of questionnaire completion. A third of our study sample (32·9%) was born in the U.S. We also had individuals self-identified as Asian immigrants who were born in China (22·9%), India (18·8%), Vietnam (18·8%), The Philippines (15·9%), or other countries (23·5%) such as Burma, Canada, France, Germany, Indonesia, Japan, Korea, Malaysia, Pakistan, Taiwan, and Thailand. For those foreign-born, the mean age at the time of immigration was 17 years old. Most of the study participants spoke English at home (70·02%), with 27·3% speaking Chinese languages (e.g., Mandarin and Cantonese), 20·4% Indian Subcontinent languages (e.g., Hindi, Gujarati, Urdu), 16·4% Vietnamese, 9·8% Filipino or Tagalog, and 3·6% speaking other Asian languages.

Among the respondents, 6·7% reported having been diagnosed with cancer. Current smokers represented 5·5% of our study sample. Among the 224 individuals answering the question related to their familiarity with AN use, 140 (90·9%) could recognize names and/or pictures of at least one AN product. An important proportion of respondents (42·6%) were not aware of any health consequences associated with AN consumption. Almost a third of the study participants (30·7%) had family members or friends living in the U.S. who use AN products. Among the 147 individuals who completed the question about AN consumption in the US, 17·1% self-reported ever use of AN products. The mean age of AN initiation was 15 years old.

Statistically significant differences in education, birth country, age at US immigration, familiarity with AN products, having friends and/or family members in the US using AN products, and being AN ever-user were found between the ISID and non-ISID subgroups. For more details about the characteristics of our study sample, please refer to Table 1.

**Table 1:** Characteristics of the Asian immigrants and descendants participating in the study (*N* = 275)^a^

Characteristics	Total *N* = 275 (100%)	Indian subcontinent Immigrant or descendant *n* = 58 (21.1%)	Others *n* = 217 (78.9%)	*p* value
**Age (*n* = 267)**				0.1271
18–34 years	88 (32.96)	13 (22.81)	75 (35.71)	
35–54 years	115 (43.07)	26 (45.61)	89 (42.38)	
≥ 55 years	64 (23.97)	18 (31.58)	46 (21.90)	
**Gender (*n* = 266)**				0.5022
Female	155 (58.27)	31 (54.39)	124 (59.33)	
Male	111 (41.73)	26 (45.61)	85 (40.67)	
**Level of Education (*n* = 275)**				**0.0329**
High school or less	56 (20.36)	6 (10.34)	50 (23.04)	
Higher than high school	219 (79.64)	52 (89.66)	167 (76.96)	
**Employment Status (*n* = 263)**				0.0876
Unemployed^b^	76 (28.90)	21 (38.18)	55 (26.44)	
Employed^c^	187 (71.10)	34 (61.82)	153(73.56)	
**US Born (*n* = 255)**				**0.0100**
Yes	84 (32.94)	11 (18.97)	73 (37.06)	
No	171 (67.06)	47 (81.03)	124 (62.94)	
**Age at US immigration**^d^ **(*n* = 179)** **(mean ± SD**^e^**)**				**0.0057**
	16.48 ± 16.03	22.72 ± 14.71	14.75 ± 16.00	
**History of Cancer diagnosis (*n* = 255)**				0.2781
Yes	17 (6.67)	2 (3.51)	15 (7.58)	
No	238 (93.33)	55 (96.49)	183 (92.42)	
**Current Smoking**^f^ **(*n* = 273)**				0.9256
Yes	14 (5.51)	3 (5.26)	11 (5.58)	
No	240 (94.49)	54 (94.74)	186 (94.42)	
**Familiarity with AN products**^g^ **(*n* = 154)**				**0.0073**
Yes (Seen at stores, home, online, etc.)	140 (90.91)	49 (100.0)	91 (86.67)	
No (I have not seen AN products)	14 (9.09)	0 (0.00)	14 (13.33)	
**Awareness of the harms associated with AN use**^**h**^** (*n* = 148)**				**0.0002**
Yes	85 (57.43)	35 (81.40)	50 (47.62)	
No	63 (42.57)	8 (18.60)	55 (52.38)	
**Have friends/family members in the US who ever use AN(*n* = 140)**				**0.0005**
Yes	43 (30.71)	23 (50.00)	20 (21.28)	
No	97 (69.29)	23 (50.00)	74 (78.72)	
**Ever use of AN products in the US**^i^ **(*n* = 123)**				**0.0008**
Yes	21 (17.07)	14 (32.56)	7 (8.75)	
No	102 (82.93)	29 (67.44)	73 (91.25)	
**Age of AN initiation (mean, SD ±)**^**j**^** (*n* = 10)**				NA
	15.40 ± 7.49	15.44 ± 7.94	15.00 ± NA	

^a^Due to missing data not all the variables add up to the total sample (*n* = 275)

^b^Unemployed/Student/Homemaker/Retired/Other

^c^Employed/Self-employed

^d^Only among those respondents not born in the US

^e^*SD* Standard Deviation

^f^Current smokers: Those who responded *'Yes'* to the questions: *'Have you smoked at least 100 cigarettes (5 packs) in your entire life?*' and *'Do you now smoke every day or some days?'*

^g^One hundred twenty-one individuals were excluded from this analysis as they chose the option *“Refuse to answer to”* or did not respond the questions *“Are you familiar with AN products?”*

^h^One hundred twenty-seven individuals were excluded from this analysis as they chose the option *“Refuse to answer”* to the question *“Are you aware of the harmful effects related to AN use?”*

^i^Twenty-four individuals were excluded from this analysis as they chose the option *“Refuse to answer”* to the question *“While in the US, have you ever used any AN product yourself?”*

^j^Only among those respondents who reported AN use

Differences between AN users and non-AN users are presented in Table 2. No statistically significant differences were found between the groups, except for ISID individuals, who were more likely to use AN products (*p* < 0·0008).

**Table 2:** Proportion of areca nut product use (*N* = 123)

Characteristics	AN users *n* = 21 (17.1%)	Non-AN users *n* = 102 (82.9%)	*p* value
**Age**			0.8592
18–34 years	8 (40.00)	35 (34.65)	
35–54 years	8 (40.00)	47 (46.53)	
≥ 55 years	4 (20.00)	19 (18.81)	
**Gender**			0.5374
Female	10 (47.62)	55 (55.00)	
Male	11 (52.38)	45 (45.00)	
**Level of Education**			0.7038
High school or less	4 (19.05)	16 (15.69)	
Higher than high school	17 (80.95)	86 (84.31)	
**Employment Status**			0.4316
Unemployed	5 (26.32)	36 (35.64)	
Employed	14 (73.68)	65 (64.36)	
**US Born (*n* = 255)**			0.2538
Yes	7 (33.33)	21 (21.65)	
No	14 (66.67)	76 (78.35)	
**Current Smoking**			0.7062
Yes	2 (10.53)	8 (7.92)	
No	17 (89.47)	93 (92.08)	
**Ancestry**			
Indian Subcontinent descendant/immigrant	14 (66.67)	29 (28.43)	**0.0008**
Other	7 (33.33)	73 (71.57)	

The number of cases in the regression model was reduced to 140 due to missing data. Results of the logistic regression that modeled AN ever use in association with ancestry (main predictor) adjusting for age range, gender, place of birth, level of education, employment status, smoking status, and having friends and/or family members in the US using AN products, showed that compared to non-ISID individuals, ISID participants were significantly more likely to be AN ever-users (OR = 3·9; 95% CI: 1·10–13·81; *p* = 0·035). Participants who had friends and/or family members using AN products were also significantly more likely to be AN ever-users themselves (OR = 6·2; 95% CI: 1·69–22·69; *p* = 0·006) (Table 3).

**Table 3:** Factors associated with areca nut use among respondents (*n* = 140)

**Independent variables**	**Odds ratio**	**95% CI**^a^	***p* value**
Ancestry, Indian Subcontinent descendant/immigrant *vs.* Other (ref^b^)	3.90	(1.10, 13.81)	**0.0351**
Age, ≥ 35 years *vs.* 34 years or younger (ref)	0.68	(0.19, 2.48)	0.5623
Gender, Male *vs.* Female (ref)	2.03	(0.54, 7.57)	0.2922
Born in US, No *vs.* Yes (ref)	0.79	(0.20, 3.16)	0.7367
Level of education, high school or less *vs.* higher than high school (ref)	1.21	(0.20, 7.42)	0.8388
Employment status, unemployed *vs.* employed (ref)	0.37	(0.09, 1.63)	0.1900
Smoking status, current smoker *vs.* non-current smoker (ref)	2.07	(0.27, 16.09)	0.4870
Family/Friends using AN products, Yes *vs.* No (ref)	6.19	(1.69, 22.69)	**0.0059**

^a^*95% CI* 95% confidence interval

^b^*ref* reference category

## Discussion

In our study, 17·1% of the participants who self-identified as Asian immigrants or descendants living in Houston reported being ever-users of AN products. This prevalence is even higher (31·2%) among individuals with Indian Subcontinent ancestry. This alarming prevalence denotes a public health concern considering that among all the Harris County Asians (6·9% of the county's inhabitants) participating in the 2010 US Census, Indians and Pakistanis represented the second-largest Asian community (20·0%) in the county [16]. Even more, between 2010 to 2017, these groups grew exponentially (Indians by 59%, and Pakistanis by 49%), resulting in India being ranked first in 2017 among the Asian origin countries of the Houston Metropolitan Area foreign-born population [4, 19].

Surprisingly, 6·7% of the respondents reported being diagnosed with cancer. This finding could be explained by the fact that our sampling strategy was supported by the outreach program of a major cancer center in Houston and/or by the concurrent use tobacco products, frequently reported by AN users [20]. While only 5·3% of the respondents using AN products reported being current smokers, our pilot study did not collect information about other cancer-risk factors.

Based on sample size and study designs, our findings can only be comparable to two reports about AN consumption in the US. These reports by Changrani et al*.*^*13*^ and Shi et al*.*^*15*^may have shown higher AN prevalence because the targeted populations were first-generation Asians migrating to the US, while our study included not only Asian immigrants but also their descendants. A research effort conducted in the Mariana Islands [21], one of the US Affiliated Pacific Islands, reported a 5-year (2011–2015) AN use prevalence of 11·3% among 300 surveyed adults residing in Guam and Saipan. This result is comparable to the 17·1% AN use prevalence reported in our study. AN chewing has also been reported among Asian groups residing in other industrialized countries. Findings from a study conducted in a large concentration of Bangladeshis living in the London boroughs of Tower Hamlet and Camden in the United Kingdom (UK) have shown an AN use prevalence ranging between 25–75% and 14–43% among adults and children, respectively [22]. Another research effort conducted among 143 first- and second-generation Bangladeshi women residing in London, UK, found that the AN use prevalence ranged between 25 and 33% without significant difference between generations [23]. Areca nut chewing was also reported in two dental patients in Australia who migrated from Myanmar and Sri Lanka [24].

Among our survey respondents, the mean age of AN initiation was 15·4 years (SD ± 7·44). This finding should be considered while designing educational programs and cessation interventions, as AN initiation during early adolescence has been shown to be associated with greater AN consumption, high levels of dependence, and a lower cumulative probability of quitting [25, 26].

Data from this population-based pilot study suggest that Indian Subcontinent immigrants or descendants (ISID) were almost 4-times more likely to be AN ever-users than Non-ISID, highlighting the need of prioritizing the AN prevention and control interventions to this particular group in Houston. Additionally, study participants who had friends and/or family members using AN products were 6 times more likely to be AN ever-users themselves. This is consistent with the results from studies conducted in the Mariana Islands, where witnessing regular AN consumption by family members and friends was among the cited reasons contributing to AN consumption among study participants [21, 27]. These findings resonate with the fact that for some Asian groups, AN consumption has a strong social connotation and cultural roots, as it is offered to guests at celebrations, weddings, and gatherings or as part of traditional beliefs [28]. Therefore, developing culturally relevant programs that serve the targeted population should be guaranteed, in addition to considering social network interventions in all efforts aiming to prevent and control AN consumption.

Although the Food and Drug Administration (FDA) banned the import of AN products into the US owing to its adulterated, unsafe nature, and carcinogenic properties; and that the US Department of Agriculture has reinforced a prohibition against the introduction of AN in raw or unprocessed forms into the US [8, 29], these products continue to be easily available across the country, mainly at Asian stores [14]. As of 2020, the US is the largest importer (59·39%) and India is the largest exporter (46·29%) of AN. The top export flow of AN in 2020 was from India to the US, with an export value of USD 2.16 million [30]. A study conducted in 5 stores selling AN products in Richmond, Virginia, revealed that the AN products were from either India or Pakistan [14]. Some importers also use misleading names for these products, such as “fragrant wood slice,” to avoid Customs scrutiny [8]. AN is also widely sold online [10] with many AN retailers utilizing the Internet and social media platforms to increase their products’ visibility, which has long been a tobacco industry strategy [31]. This was also a purchase avenue pursued by some of our survey respondents. Unfortunately, there are no federal or state laws that explicitly restrict, monitor, and/or regulate AN products’ online marketing in the US. As it already happens with tobacco products, particularly among youth [32], Internet AN sales may pose an attractive route for many consumers seeking cheaper, tax-free products. Therefore, such easy accessibility to AN products may be responsible for the prevalence of AN use in the US. Regulatory agencies in the US could develop and implement similar regulations to those recently imposed by the Food Safety and Standards Authority of India (FSSAI), where all imported consignments of AN entering to this country undergo 100% sampling and testing, as opposed to merely random checks [33]. However, this will require implementing tailored training programs aiming at building capacity of FDA officials and Custom agents. India has also raised AN minimum import price (MIP: rate below which no imports are allowed) by almost three times in recent years to deter AN imports into the country [34]. In the US, either banning the sales of AN products (action already taken by Canada) [5] or implementing effective national control laws similar to those imposed on tobacco products (e.g., pictorial health warning on AN products, banning AN sales to minors, AN-free policies on public spaces, among others) should be considered towards preventing AN addiction and decrease the risk of oral cancer and other detrimental conditions related to AN use. Regarding implementing effective national AN control laws, the WHO Framework Convention on Tobacco Control (FCTC) [35] has established a package of standardized and validated measures for eliminating illicit trade of tobacco products that could be adapted to AN products.

Even though prolonged AN consumption is associated with a myriad of adverse health effects such as tooth decay; periodontitis; OPMDs; oral/pharyngeal, esophageal and hepatocellular cancers; liver cirrhosis; metabolic syndromes likely contributing to type 2 diabetes mellitus; hypertension; cardiovascular disease; anemia; pregnancy-related disorders; asthma; and chronic kidney disease [15, 36, 37], a large proportion of individuals in our study sample (42·6%) were unaware of the adverse health effects of AN use. This result contrasts with what was reported by a study conducted in Karachi, Pakistan, among 370 subjects where more than 70% of these individuals acknowledged that AN chewing might contribute to oral/oropharyngeal cancers [38]. Similarly, there was a high awareness (80%) of cancer risk among 200 AN users recruited in a study conducted in Yangon, Myanmar [4]. The lower awareness of the harmful effects of AN consumption among our study sample underscores the need for health promotion programs in the Houston Metropolitan Area focusing on the broad spectrum of short-term and long-term deleterious health effects caused by AN use.

Healthcare providers (HCPs), especially dental professionals, can play a crucial role in identifying the oral clinical signs of AN use [39]. Chronic AN use can have potentially deleterious effects on both hard and soft tissues of the oral cavity. The hard, fibrous nature of the AN may cause tooth fracture and extensive attrition of the teeth [39]. Also, the copious red saliva produced on AN chewing gets embedded into and stains the teeth, gingiva, and oral mucosa [40]. In chronic chewers, a condition called betel chewers mucosa is often found where the quid is placed, characterized by oral mucosa desquamation and underlying pseudomembranous or wrinkled areas [39]. AN consumption can also lead to several OPMDs, such as oral submucous fibrosis (OSMF), leukoplakia, and lichenoid lesion. AN is the primary etiological factor in the development of OSMF. This condition is usually preceded by the formation of vesicles, followed by increased fibrosis that leads to stiffness and diffuse blanching of the oral mucosa, trismus, and reduced tongue protrusion [41]. AN chewing can also lead to leukoplakia—a predominantly white patch or plaque on the oral mucosa, which has an increased risk of malignant transformation [39]. Betel quid‑induced lichenoid oral lesions have been reported exclusively among AN users at the quid placement sites and have the potential to progress to oral cancer. This oral condition is characterized by fine, white, wavy, non-overlapping, parallel lines that in some instances radiate from a central erythematous area [39]. Hence, educational programs can strengthen the capacity of HCPs to screen for AN use and early diagnosis and treatment of conditions associated with AN consumption in the ISID population. Unfortunately, specialized AN cessation services and resources are lacking, which negatively impact healthcare providers’ motivation in assisting AN users with their quitting efforts. Perhaps the critical step for promoting AN prevention and control strategies should be to develop the curriculum of a training program based on stages of change [42], motivational interviewing techniques [43] and the 5As intervention (Ask, Advise, Assess, Assist, and Arrange) [44]. These approaches, developed initially for smoking cessation, can be adapted to provide practitioners with a framework for AN counseling, as this model has proved to be successful in achieving long-term abstinence with other additions (.e.g., smoking cessation) and developing strategies for early diagnosis of conditions associated with AN consumption.

**Fig. 1:**
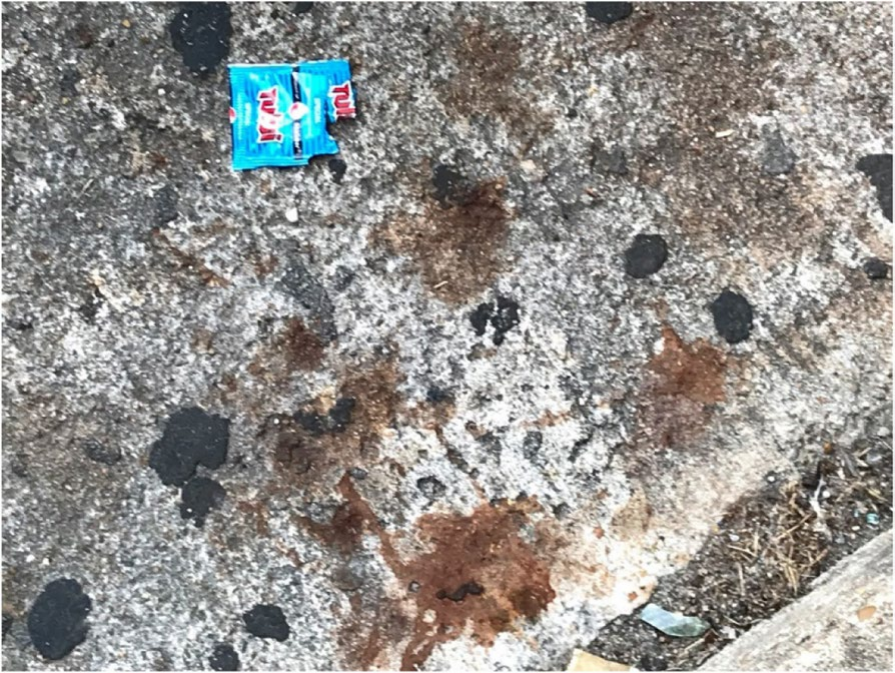
Evidence of AN spitting on the pavement of a parking lot located in an Asian neighborhood in Houston, Texas

**Fig. 2:**
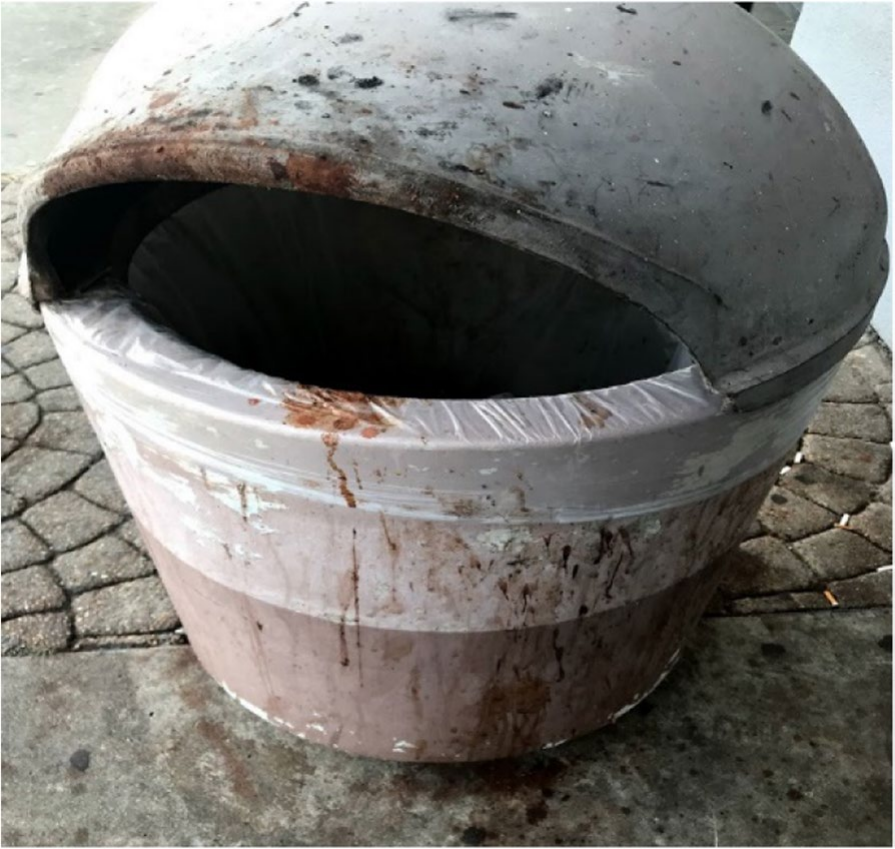
Trash receptacle splattered by AN spittle

The recent COVID-19 pandemic has raised concerns that AN consumption, like smokeless tobacco use, may increase risk of virus transmission. Since AN users spit frequently, droplets from COVID-19 infected chewers can settle on objects and surfaces that can be touched by non-infected individuals (Figs. 1 and 2). The transmission risk of several viral and some non-viral diseases through saliva is well documented [45]. So, in the same way, that covering one’s cough or sneeze is recommended to prevent disease transmission, avoiding spitting, especially in public venues, would seem a logical extension of these recommendations [46]. This becomes even more relevant during the ongoing COVID-19 pandemic.

Although our study is among the five research efforts focusing on AN use in the US mainland in the past 25 years, several limitations must be considered. The main caveat is that this study was based on cross-sectional data. Therefore, we cannot assume any causal relationships between AN use and oral lesions or systemic conditions. Due to the nature of our pilot study and limited resources available, we did not validate the questionnaire items. Despite that, our findings set the foundation for future mixed-methods approaches with content experts and AN users to measure the validity and reliability of the proposed data collection instrument and use it to assess AN consumption in a larger sample of Asian groups residing in Houston and other large cities in Texas. Also, our findings are not truly representative of the entire Asian population in Houston. Surveyed participants were selected using non-probability sampling methods, reaching out to attendees of health fairs and cultural events in the area (selection bias). Nevertheless, our study population included a balanced proportion of the main Asian groups residing in Houston. In addition, missing data could result from study participants’ perceiving sensitivity towards some of the research questions in our data collection instrument, particularly those related to AN consumption. Also, the study design used self-reporting, which makes the research findings prone to subjectivity (information bias). Finally, social desirability bias could be expected, as some of the responses may not reflect the true thoughts of our study participants.

## Conclusions

Based on the alarming AN use prevalence, mainly among ISID participants in our study, a more profound understanding of the different cultural practices and beliefs associated with AN use among this particular group is required. Therefore, future research efforts with larger sample size are needed to better understand the true impact of AN use in the US mainland. This would enable the delivery of appropriate services, including AN-associated health risk awareness and educational programs, as well as AN cessation interventions tailored to this vulnerable segment of the Asian population.

## Data Availability

The datasets used and/or analyzed during the current study are available from the corresponding author on reasonable request.

## Abbreviations

AN: Areca Nut
CI: Confidence Intervals
COVID-19: Coronavirus infection
FCTC: Framework Convention on Tobacco Control
FDA: Food and Drug Administration
HCP: Healthcare Provider
FSSAI: Food Safety and Standards Authority of India
ISID: Indian Subcontinent Immigrants or Descendant
MIP: Minimum Import Price
OSMF: Oral Submucous Fibrosis
OPMD: Oral Potentially Malignant Disorders
OR: Odds Ratio
SD: Standard Deviation
UK: United Kingdom
UN: United States
WHO: World Health Organization
